# Characterizing the Impact of Chitosan on the Nucleation and Crystal Growth of Ritonavir from Supersaturated Solutions

**DOI:** 10.3390/polym15051282

**Published:** 2023-03-03

**Authors:** Arif Budiman, Kalina Kalina, Levina Aristawidya, Adnan Aly Al Shofwan, Agus Rusdin, Diah Lia Aulifa

**Affiliations:** 1 Department of Pharmaceutics and Pharmaceutical Technology, Faculty of Pharmacy, Universitas Padjadjaran, Jl. Raya Bandung-Sumedang Km. 21, Bandung 45363, Indonesia; 2 Department of Pharmaceutical Analysis and Medicinal Chemistry, Faculty of Pharmacy, Universitas Padjadjaran, Jl. Raya Bandung-Sumedang Km. 21, Bandung 45363, Indonesia

**Keywords:** supersaturation, polymer, amorphous, chitosan, nucleation, crystallization, ritonavir, low crystallization tendency

## Abstract

The addition of polymeric materials is often used to delay nucleation or crystal growth and maintain the high supersaturation of amorphous drugs. Therefore, this study aimed to investigate the impact of chitosan on the supersaturation behavior of drugs with a low recrystallization tendency and elucidate the mechanism of its crystallization inhibition in an aqueous solution. It was carried out using ritonavir (RTV) as a model of poorly water-soluble drugs categorized as class III of Taylor’s classification, while chitosan was used as a polymer, and hypromellose (HPMC) was used for comparison. The inhibition of the nucleation and crystal growth of RTV by chitosan was examined by measuring the induction time. The interactions of RTV with chitosan and HPMC were evaluated by NMR measurements, FT-IR, and an in silico analysis. The results showed that the solubilities of amorphous RTV with and without HPMC were quite similar, while the amorphous solubility was significantly increased by the chitosan addition due to the solubilization effect. In the absence of the polymer, RTV started to precipitate after 30 min, indicating that it is a slow crystallizer. Chitosan and HPMC effectively inhibited the nucleation of RTV, as reflected by a 48–64-fold enhancement in the induction time. Furthermore, NMR, FT-IR, and in silico analysis demonstrated that the hydrogen bond interaction between the amine group of RTV and a proton of chitosan, as well as the carbonyl group of RTV and a proton of HPMC, was observed. This indicated that the hydrogen bond interaction between RTV and chitosan as well as HPMC can contribute to the crystallization inhibition and maintenance of RTV in a supersaturated state. Therefore, the addition of chitosan can delay nucleation, which is crucial for stabilizing supersaturated drug solutions, specifically for a drug with a low crystallization tendency.

## 1. Introduction

The improvement of drug solubility is very important in the development of orally administered formulations because almost 80% of new drug candidates have poor water solubility [1,2]. The aqueous solubility of a drug also influences its bioavailability due to absorption after dissolving in the gastrointestinal fluid [3,4]. Drug amorphization is one of the strategies that is commonly used to improve the solubility of a poorly water-soluble drug [5]. The amorphous drug has a higher free energy compared to its crystalline state and can form supersaturated solutions in an aqueous medium [6]. However, the crystallization of poorly water-soluble formulations in the supersaturated state is due to their thermodynamic instability in supersaturated aqueous solutions. This makes the addition of excipients very important to inhibit the crystallization of a poorly water-soluble drug in a supersaturated aqueous solution [7].

The addition of polymeric materials has commonly been used in the formulation of a supersaturated drug to delay crystallization and maintain the high supersaturation of amorphous drugs [8,9]. However, the tools used to predict the rational selection of the optimal polymer for maintaining supersaturated solutions of drugs are still scarce. Crystallization inhibition and the maintenance of supersaturated systems of drugs with polymer addition depend on their ability to inhibit nucleation and subsequent crystal growth. This makes it necessary to understand the fundamentals of nucleation kinetics and the mechanism of inhibition by polymers to optimize the crystallization inhibition of drugs in supersaturated solutions [7].

Various polymers have been studied to inhibit drug crystallization in supersaturated solutions and maintain high concentrations for extended periods. These include hydrophilic polymers, such as methacrylate copolymers [10], hypromellose acetate succinate (HPMC-AS) [11], polyvinylpyrrolidone (PVP) [12], and hypromellose (HPMC) [13], which effectively inhibit drug crystallization in supersaturated solutions of drugs [14]. These polymers are commonly used in amorphous solid dispersions to achieve higher drug supersaturation for oral administration. A previous study established that drug–polymer interactions and molecular mobility suppression play a significant role in the inhibition of drug recrystallization in supersaturated solutions [15].

**Figure 1:**
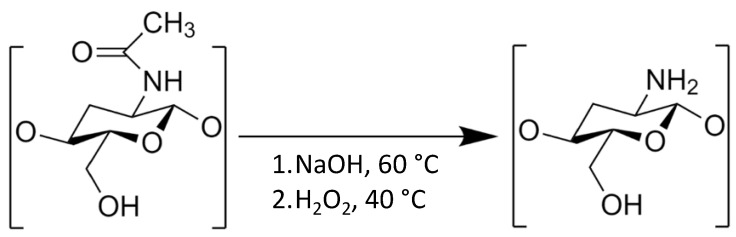
Deacetylation process of chitin. Adapted from data originally presented in Ref. [22].

Chitosan, derived from chitin, is a potential polymer derived from naturally occurring sources that can be used in pharmaceutical formulations. It is a linear copolymer of (1–4)-linked 2-acetamido-2-deoxy-d-glucopyranose and 2-amino-2-deoxy-d-glycopyranose produced by the alkaline N-deacetylation of chitin [16,17]. Chitin is found in fungal sources, aquatic sources, and terrestrial sources, such as the cell walls of crustaceans, fungi, and insects [18,19]. Since chitin is insoluble in aqueous solutions and organic solvents, deacetylation should be conducted to enhance its solubility, which is more suitable for useful bioapplications (Figure 1). Chitosan can be prepared by various methods, such as solvent casting, compression molding, freeze drying, electrospinning, and the dense gas foaming method [19]. Chitosan can be modified with other bioactive molecules to impart additional properties to the tissue construct. Moreover, chitosan can also facilitate three-dimensional cell growth and proliferation as well as organize the deposition of collagen, which ensures rapid healing [20]. Commonly, membranes/films, hydrogels, sponges, and fiber forms of chitosan have been widely used for wound healing or tissue engineering applications [21]. Because it is a biocompatible, biodegradable, and nontoxic polymer, chitosan is an important material that can be used in a variety of biomedical domains, including pharmaceutical formulations [7,17]. Therefore, chitosan can form supersaturated solutions in amorphous drug formulations after dissolving in aqueous solutions.

A previous study reported the crystallization inhibition of a drug using water-soluble chitosan as a polymer [7]. The model drug used was alpha-mangosteen, with a high recrystallization tendency. Meanwhile, the elucidation of the crystallization inhibition mechanism of a drug with a low recrystallization tendency using chitosan as a polymer has not been investigated, particularly for an amorphous drug categorized as class III of Taylor’s classification [23]. The mechanism of crystallization inhibition for each drug will be different due to differences in the individual’s specific physicochemical properties [9]. Therefore, this study aimed to evaluate the inhibition mechanism of crystal nucleation by chitosan for a drug with a low crystallization tendency based on a molecular-level characterization in a supersaturated solution. The impact of hypromellose (HPMC) on the inhibition mechanism of the drug was also examined for comparison. Ritonavir (RTV) was selected as a model of poorly water-soluble drugs because of its low recrystallization tendency and categorization in class III. Although some drugs with a low recrystallization tendency were stable after cooling and reheating, their thermodynamical instability led to nucleation and crystal growth, specifically after dispersion in water. This makes it crucial to investigate the ability of chitosan to inhibit the crystallization of RTV in supersaturated solutions to determine whether the polymer is needed for the improvement of the pharmaceutical properties of drugs.

## 2. Materials and Methods

### 2.1. Materials

**Figure 2:**
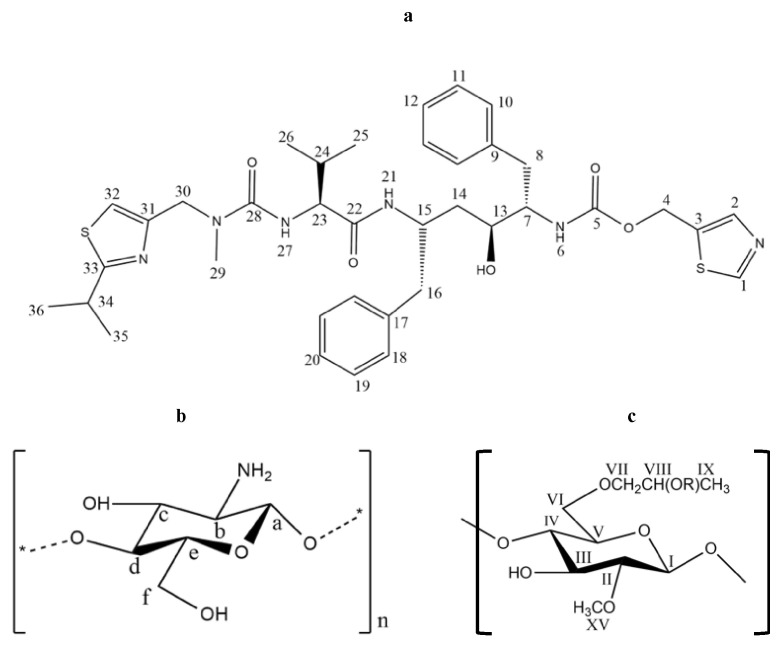
Chemical structures of (**a**) RTV, (**b**) chitosan, and (**c**) HPMC.

RTV (MW = 720.95 g/mol) was purchased from ChemShuttle (Hayward, Berkeley Heights, NJ, USA), while chitosan and HPMC were purchased from Biochitosan Indonesia (Cirebon, Indonesia) and Merck (Darmstadt, Germany), respectively. Their chemical structures are represented in Figure 2.

### 2.2. PXRD Measurement

The PXRD patterns of each sample were collected by using a Kristalloflex Diffractometer (D500, Siemens, Berlin, Germany) with the following conditions: target Cu, voltage 40 kV, filter Ni, current 30 mA, scanning rate 0.75°/min, and scanning angle of 2θ = 10°–40°.

### 2.3. Preparation and Evaluation of Amorphous RTV by Melt Method Using DSC

The RTV crystal was placed into a crimped aluminum DSC pan, heated to 140 °C with a heating rate of 10 °C/min, and held for 10 min. Subsequently, the samples were quenched until −20 °C was reached, and calorimetric analysis was conducted by heating amorphous RTV using a heating rate of 10 °C/min.

### 2.4. Crystalline and Amorphous Solubility Determination

The crystalline solubility of RTV was determined using water containing 3% (*v*/*v*) methanol, while chitosan was dissolved in water at various concentrations. This was followed by the addition of excess crystalline RTV powder to the chitosan solution and shaking for 48 h at 25 °C. Subsequently, the solutions were filtered through a 0.45 μm membrane filter, diluted with acetonitrile, and analyzed using high-performance liquid chromatography (HPLC). The amorphous solubility of RTV was also determined using centrifugation, as described in a previous report by Dening et al. (2018) [24].

### 2.5. HPLC Conditions

HPLC was analyzed using a Dionex-Ultimate 3000 HPLC (Dionex, Sunnyvale, CA, USA) equipped with an Inertsil ODS C18 (6.0 × 150 mm) column at 35 °C. The mobile phase was composed of acetonitrile and water at a ratio of 7:3, with a flow rate of 0.7 mL/min. The samples were analyzed by injecting 10 μL at 244 nm using a UV detector. Standard solutions containing 1, 5, 10, 25, 50, and 100 μg/mL RTV in acetonitrile were diluted with water, filtered through a 0.45 μm membrane filter, and analyzed using HPLC. The calibration curves were plotted as the peak area of RTV versus the amount of each analyte. The linearity was evaluated by linear regression analysis calculated using the least-squares regression method. The linear coefficient of determination (R^2^) value of the RTV standard solution used was 0.9998. The experiment was performed in triplicate (mean values + SD).

### 2.6. Nucleation Induction Time Measurements

Each polymer (chitosan and HPMC) was dissolved in an aqueous solution at a concentration of 500 μg/mL. The supersaturated solutions were prepared by adding a stock solution of RTV (1500 μg/mL in methanol) to the polymer solutions, leading to a final methanol concentration of 2% (*v*/*v*) at 25 °C. The solutions were stirred at 25 °C, filtered through a 0.45 μm membrane filter at different time points, diluted with acetonitrile, and determined using HPLC.

### 2.7. Fourier Transform Infrared (FT-IR)

The supersaturated solutions of the samples used in nucleation induction time measurements were evaluated using a Nicolet iS5 FT-IR spectrometer (Thermo Scientific, Waltham, MA, USA). This was carried out to determine the interaction between RTV and the polymer in an aqueous solution, as described in a previous report [7,15].

### 2.8. NMR Measurements

Each polymer was dissolved in methanol-d4, and supersaturated solutions of RTV were prepared by adding a stock solution of RTV in methanol-d_4_ to the polymer solutions, yielding a final methanol-d_4_ concentration of 3% (*v*/*v*) at 25 °C. The sample solution was measured after stirring at 150 rpm at 25 °C for one minute and immediately transferred to a 5 mm NMR tube for NMR measurements using Bruker 500 MHZ NMR spectrometer (Billerica, MA, USA).

### 2.9. In Silico Study

The interactions between RTV and chitosan were examined in an in silico study using a computer, as described in a previous report [7,15]. This was carried out to obtain the hydrogen bonding, binding energy, and distance of each interaction between RTV and chitosan.

### 2.10. Viscosity Test

Viscosity was measured using an Ametek Brookfield DVE viscometer. The size of the spindle used was No. 61, with a rotation speed of 100 rpm.

**Figure 3:**
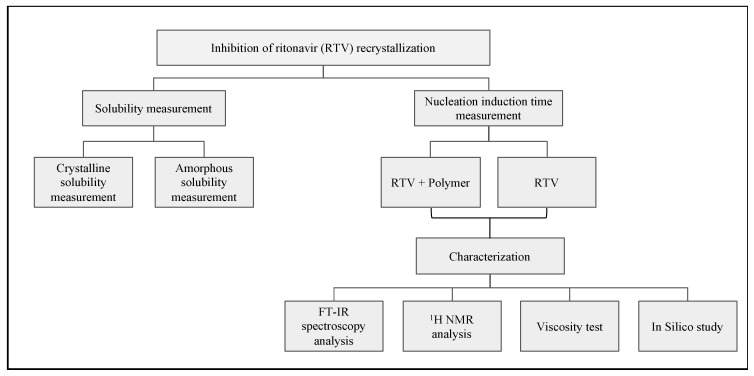
Flow chart of the methodology.

A flow chart of the methodology is shown in Figure 3.

## 3. Results

### 3.1. PXRD Measurement

**Figure 4:**
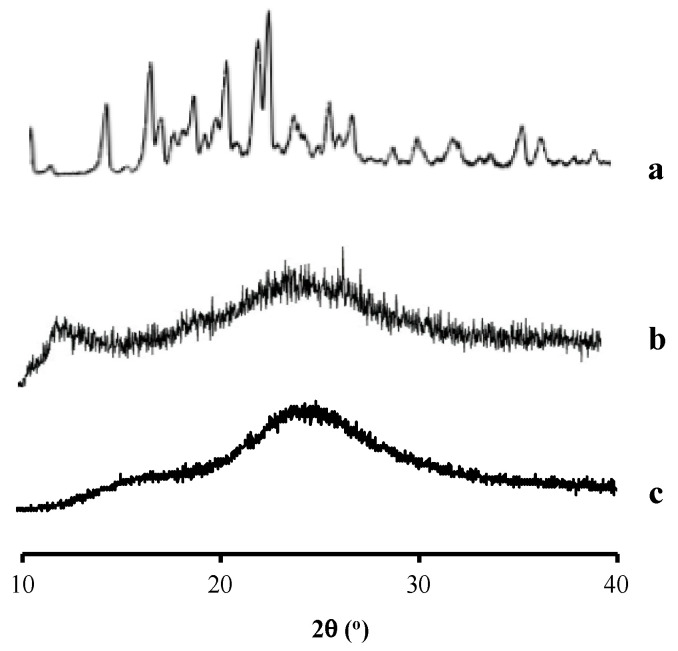
Characteristic diffraction peaks in PXRD of (**a**) RTV, (**b**) chitosan, and (**c**) HPMC.

The RTV sample showed characteristic diffraction peaks in the PXRD pattern, indicating its crystalline state, and the peaks were similar to those of RTV crystal form I [25,26], as shown in Figure 4. This indicated that the starting material of RTV used in this study was a form I polymorph, which is the thermodynamically stable one [27]. Meanwhile, chitosan and HPMC had a halo pattern without any diffraction peaks, indicating that they were in an amorphous state.

### 3.2. Preparation and Evaluation of Amorphous RTV by Melt Method Using DSC

**Figure 5:**
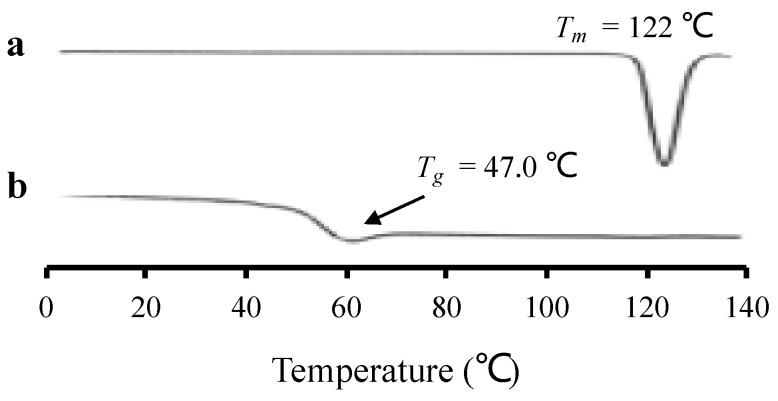
DSC curve of (**a**) RTV crystal (first heating), and (**b**) amorphous RTV (second heating).

Figure 5 shows the DSC curve of amorphous RTV prepared by the melt quenching method to confirm the recrystallization tendency of RTV. In the first heating, RTV showed a melting peak at 122 °C, indicating its crystalline state. Meanwhile, in the second heating after the cooling process, a glass transition event was observed at 47.0 °C, without a melting peak. This implies that amorphous RTV has a low recrystallization tendency, indicating it does not crystallize upon reheating, as commonly observed for drugs categorized as class III of Taylor’s classification.

### 3.3. Crystalline and Amorphous Solubility Determination

**Figure 6:**
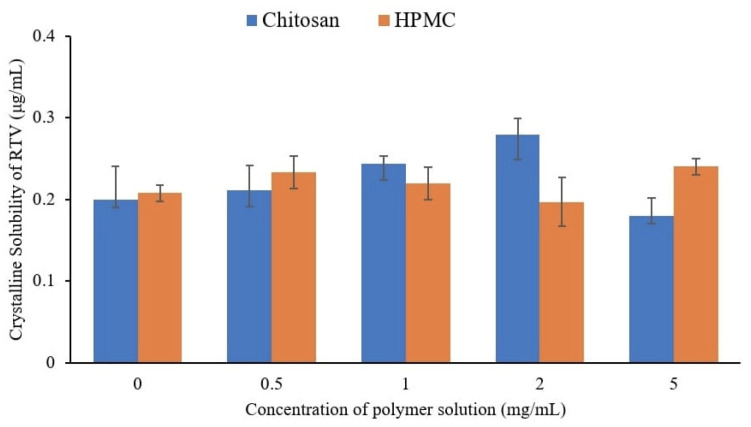
Crystalline solubility of RTV at 25 °C in polymer solutions with various concentrations.

To evaluate the effect of the polymer on the crystalline solubility of RTV, the equilibrium solubilities of AM in the absence and presence of selected polymers with various concentrations were determined, as shown in Figure 6. The equilibrium solubility of crystalline RTV in water at 25 °C was 0.22 ± 0.04 μg/mL. This indicates that the RTV crystal has extremely poor aqueous solubility. A previous investigation stated that the presence of polymers can increase the equilibrium solubility of a drug [14]. In this study, the presence of chitosan and HPMC did not affect the thermal equilibrium solubility of the RTV crystal in the water at 25 °C. Therefore, the effect of the polymers (chitosan and HPMC) on the equilibrium solubility of RTV can be considered negligible. The lowest polymer concentration (500 μg/mL) was further used for the induction time measurement.

Table 1 shows the solubilities of the RTV crystal and its amorphous form in water with and without the polymers at a concentration of 500 μg/mL. The presence of chitosan and HPMC at a concentration of 500 μg/mL did not change the equilibrium solubility of crystalline RTV, but it was significantly increased by the chitosan addition. This is due to drug solubilization caused by adding chitosan that occurred in amorphous RTV, leading to a reduction in the solute thermodynamic activity of RTV [28,29]. The concentration-based supersaturation ratio of RTV (S_a,RTV_/S_c,RTV_) also increased in chitosan solutions. Meanwhile, the solubility in the HPMC solution was not significantly increased compared to the value observed for water. The concentration-based supersaturation ratio of RTV (S_a,RTV_/S_c,RTV_) was almost similar between water and the HPMC solution.

### 3.4. Nucleation Induction Time Measurements

**Figure 7:**
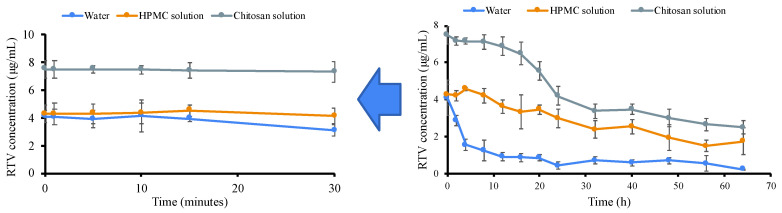
(**left**) Expanded (for 30 min) and (**right**) full graphic of RTV concentration in water and each polymer solution (*n* = 3, mean ± standard deviation).

The crystallization tendency of each drug from the solution can be represented by its nucleation induction time. Figure 7 shows the induction time determined by plotting the concentrations of RTV in the supersaturated solutions versus time with and without the presence of polymers (500 μg/mL). The initial RTV concentration in each sample was set at its amorphous solubility, which was approximately 19–32 times higher than the 0.21 μg/mL crystalline solubility. The initial concentrations of RTV with and without HPMC are quite similar, indicating that the rate of crystallization of the dissolving amorphous solid from the supersaturated solution is relatively slow. Moreover, the high concentration of RTV in water can be maintained for 30 min, which gradually decreases until reaching the equilibrium solubility level of the RTV crystal after 24 h. This indicates that RTV is a slow crystallizer and is classified as class III of Taylor’s classification. The high concentration of RTV in the HPMC solution was maintained for 24 h and started to decrease after 32 h. However, the concentration of RTV was still higher than its crystalline solubility after 64 h. This is due to the slow crystallization of RTV and the presence of HPMC, with a strong ability to inhibit the rapid crystal nucleation of RTV in a supersaturated solution. As shown in Table 1, the high concentration of RTV in the chitosan solution was maintained for 24 h at an amorphous solubility greater than the initial value in water and the HPMC solution but started decreasing after 24 h. Similarly, after 64 h, the RTV concentration was also still higher than its crystalline solubility. The solubilization effect of chitosan, the slow crystallization of RTV, and the intermolecular interaction between RTV and chitosan contributed to the inhibition of RTV crystal nucleation in a supersaturated solution.

### 3.5. NMR Analysis

**Figure 8:**
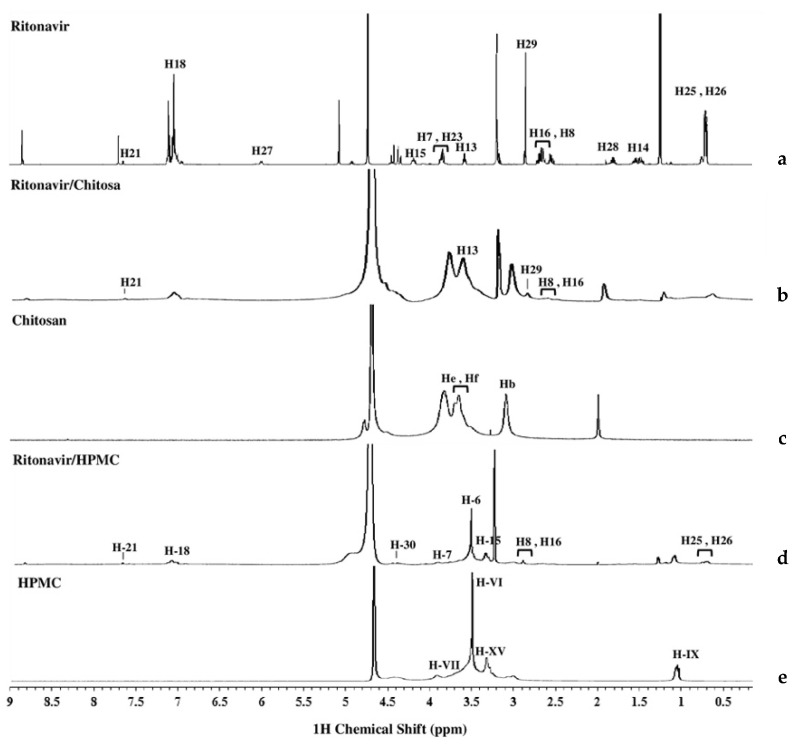
Full ^1^H NMR spectra of (**a**) RTV, (**b**) RTV-chitosan, (**c**) chitosan, (**d**) RTV-HPMC, and (**e**) HPMC peak regions in the aqueous solution.

**Figure 9:**
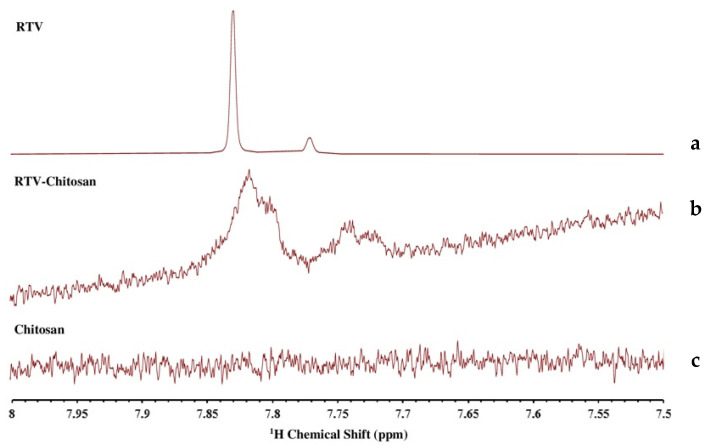
Expanded ^1^H NMR spectra of (**a**) RTV, (**b**) RTV-chitosan, and (**c**) chitosan.

^1^H NMR was performed to evaluate the interaction between RTV and the polymers (chitosan and HPMC) in the supersaturated solution. This is because the crystal nucleation of a drug in the solution occurs on a tiny scale. Therefore, molecular-level characterization is required to reveal the mechanism of drug crystal nucleation inhibition. NMR is a useful technique for the in situ characterization of the molecular states of drugs and polymers in solutions. The chemical shifts and peak widths in the NMR spectra reflect the chemical environment and molecular mobility of the compounds, which was used to evaluate molecular associations in the solution and the intermolecular interactions between the drug and polymers [14]. The ^1^H NMR spectra of the RTV-saturated solutions in the presence of HPMC and chitosan are shown in Figure 8, while the peak spectra were assigned as described in previous studies [7,30,31]. In the HPMC solution, the RTV peaks were shifted upfield by the addition of HPMC for the RTV supersaturated solutions, specifically for H16, H18, H21, and H23, which were attributed to protons from the methylene and amine groups of RTV. Moreover, the RTV peaks were significantly broadened compared to those observed in water, which were attributed to molecular mobility suppression. The peaks of HPMC were also shifted upfield by the addition of RTV, specifically for HXV, which was attributed to protons of HPMC. This suggested that protons from HPMC formed intermolecular interactions with the carbonyl group of RTV in the supersaturated solution. Similarly, the upfield shifts and peak widths of RTV peaks were observed with the addition of chitosan to supersaturated solutions, specifically for H21 and H29, which were attributed to the amine group of RTV (Figure 9). The peaks of chitosan were also shifted downfield for Hb, He, and Hf, which was attributed to a proton from the methyl group, with the addition of RTV. This suggested that the proton from chitosan formed intermolecular interactions with the amine group of RTV in the supersaturated solution, leading to mobility suppression and the long-term maintenance of RTV supersaturation. The chemical shifts and differences in each sample are summarized in Table 2.

### 3.6. FT-IR Spectroscopy Analysis

**Figure 10:**
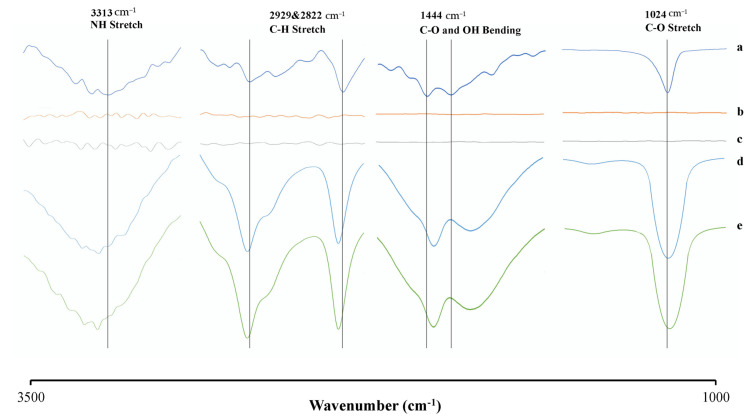
FT−IR spectra of (a) RTV, (b) chitosan, (c) HPMC, (d) RTV in chitosan solution, and (e) RTV in HPMC solution.

Solution FT-IR spectroscopy was also performed to confirm the interaction of RTV with the polymer in the supersaturated solution. It is difficult to obtain the infrared spectra of each sample in an aqueous solution because water can strongly absorb infrared radiation. Therefore, the FT-IR spectrum of water was subtracted from each sample to obtain the FT-IR spectra in an aqueous solution, as shown in Figure 10. The RTV spectra revealed peaks at 1024 cm^−1^, which was attributed to the C-O stretching group, and at 1444 cm^−1^, 2929–2822 cm^−1^, and 3313 cm^−1^, attributed to C-O and OH binding, C-H stretching, and N-H stretching, respectively. Meanwhile, chitosan and HPMC showed a low-intensity peak response at wave numbers of 2800–3300 cm^−1^ and 3300–3500 cm^−1^, which were attributed to O-H stretching, aromatic C-H, and N-H stretching, respectively.

The intensity of the spectra from RTV was increased and shifted to higher and lower values in the presence of chitosan and HPMC. The carbonyl group of RTV in the HPMC solution was shifted from 1444 cm^−1^ to 1455 cm^−1^, suggesting the possible intermolecular interaction of an acceptor of hydrogen with HPMC as a donor of hydrogen. The amine (N-H) group and aliphatic C-H groups of RTV in the chitosan solution were also shifted from 3313 cm^−1^ to 3315 cm^−1^, 2929 cm^−1^ to 2934 cm^−1^, and 2822 cm^−1^ to 2834 cm^−1^. This is due to the intermolecular interaction between the amine group of RTV as an acceptor of hydrogen with chitosan as a donor of hydrogen. These results are in line with the values obtained in the NMR measurement.

### 3.7. In Silico Study

**Figure 11:**
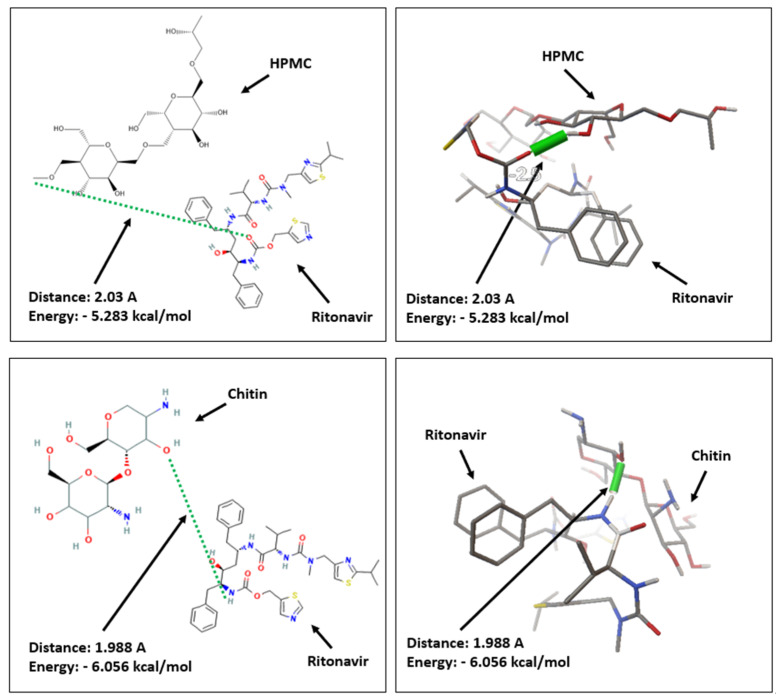
(**left**) Two−and (**right**) three-dimensional visualization of RTV with HPMC and chitosan.

Molecular docking studies were carried out to predict the interaction between ritonavir molecules and each polymer (HPMC and chitosan). The parameters used to assess the most stable polymers that have the strongest interaction with RTV include free energy binding, the bond distance, and the kind of interaction. Figure 11 reveals a hydrogen bonding interaction between RTV and HPMC, with a free energy binding of −5.283 kcal/mol and a bond distance of 2.03 Å. This occurred between the oxygen atoms of the RTV carbonyl group and the hydrogen atoms of the hydroxyl group of the HPMC polymer. In the case of chitosan, hydrogen-bonding interactions between oxygen atoms in the hydroxyl groups of the chitin polymer and hydrogen atoms in the amine groups of the RTV structure were also discovered, with a free energy binding and bond distance of −6.056 kcal/mol and 1.988 Å, respectively.

Hydrogen-bonding interactions are produced between groups of chemical compounds composed of hydrogen atoms and nitrogen (N), oxygen (O), or fluorine (F) atoms. Furthermore, hydrogen bonding is crucial in determining a molecule’s structure, characteristics, and functions. This interpretation shows that the lower the energy, the smaller the activation energy needed to make the two chemical compounds interact with one another, which will trigger a spontaneous reaction. This bond also plays a crucial role in stabilizing the interaction between the ligand and the receptor, or between the ligand and another ligand. The bond distance is used to interpret the distance of the interaction between atoms, where a closer value presents stronger binding. Moreover, the longer the bond distance that occurs, the weaker the bond is and the easier it is released.

According to molecular docking studies, the bond energy formed between RTV and the chitin polymer is lower than that between RTV and the HPMC polymer. Meanwhile, the bond distance generated by RTV and the chitin polymer is shorter than that of RTV and the HPMC polymer. This shows that the molecular docking interaction between RTV and the chitin polymer is stronger and more stable than that between RTV and the HPMC polymer.

### 3.8. Viscosity Study

The viscosity measurement of each sample was conducted because a high-viscosity solution can inhibit the recrystallization of the drug through mobility suppression. The viscosity of each sample is shown in Table 3.

Although the addition of each polymer increased the viscosity of the solution, the viscosity of HPMC and chitosan solutions slightly increased compared to water. Therefore, the difference in the viscosity of solutions with and without polymer was not significant. This indicates that the effect of increasing the viscosity on the crystallization inhibition of RTV can be neglected. The crystallization inhibition of RTV from a supersaturated solution can also be attributed to the interaction between RTV and each polymer.

## 4. Discussion

Amorphization is a strategy to improve the absorption of a drug because it can form drug supersaturation, specifically for those classified as class II or IV by the Biopharmaceutics Classification System. However, crystallization is always observed after dispersion in an aqueous solution, which is indicated by decreasing the drug concentration. This shows that the addition of polymers such as chitosan is necessary to inhibit drug crystallization and maintain the supersaturated state to enhance mass transport across a biological membrane [9,32]. The intermolecular interaction between the drug and the polymer can be a key parameter in nucleation inhibition. A previous study established that the strong affinity between the polymer and the solute can prevent the reorganization of solute clusters, leading to its recrystallization [33].

Previous studies also reported that each drug has a specific crystallization tendency upon cooling from the melt and during rapid solvent evaporation [23]. Drugs have been classified as fast (class I), intermediate (class II), or slow crystallizers (class III) based on their specific behaviors. On the basis of the crystallization tendency from the undercooled melt, ritonavir is categorized as a slow crystallizer. For drugs that have a low crystallization tendency or do not crystallize immediately, the knowledge of the amorphous solubility is needed to predict potential improvements in absorption when using an ASD formulation. The solubilities of amorphous RTV with and without the polymer addition in this study are quite similar, specifically in the presence of HPMC. This indicates that the crystallization rate of the dissolving amorphous RTV solid exposed to an aqueous solution and from a supersaturated solution is relatively slow [9].

**Figure 12:**
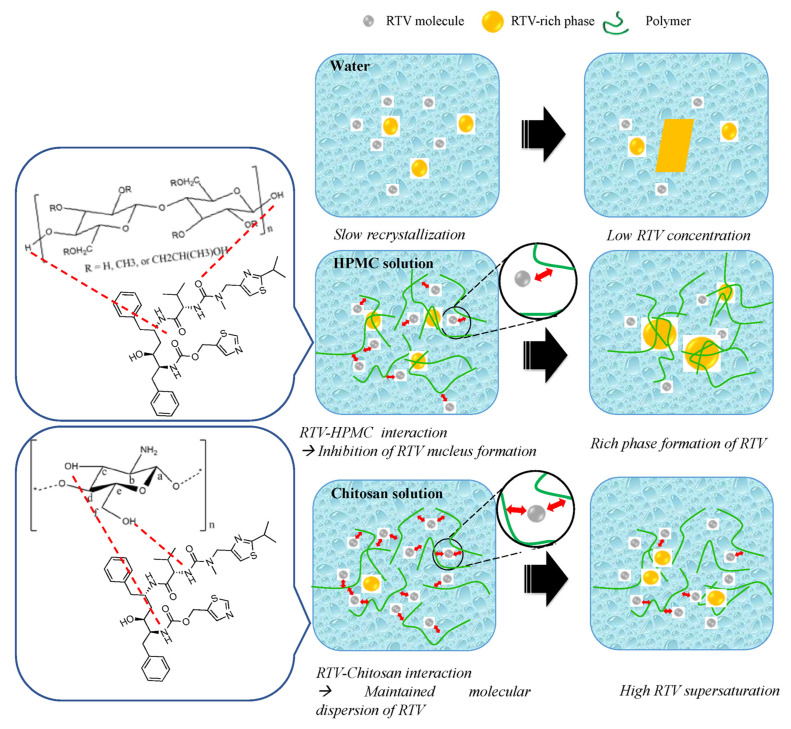
Schematic illustration of crystallization inhibition of RTV in HPMC and chitosan solutions.

The measurement of the induction nucleation time has been proposed to predict the crystal nucleation of a drug from a supersaturated solution. In this study, the mechanism of chitosan and HPMC as polymers in maintaining the supersaturation levels of RTV in a supersaturated solution is discussed, as shown in Figure 12. The amorphous solubility of RTV was found to be 4.1 μg/mL, while the induction time of RTV in the absence of the polymer was 30 min. A previous report stated that the solubility of amorphous RTV was around 27 μg/mL, and the induction time in the absence of the polymer was found after 120 min. The difference in results was due to the variation in the crystallinity of RTV and the crystalline polymorph (form I and II). Based on the induction nucleation time in Figure 6, the RTV concentration did not rapidly decrease at the beginning of the measurement and still maintained high supersaturation for 30 min. This may be due to the high molecular weight and greater conformational flexibility of RTV. This reduced the rate of nucleation of RTV from supersaturated solutions compared to drugs with a high recrystallization tendency, such as alpha-mangosteen [7]. However, the concentration of RTV gradually decreases until reaching the equilibrium solubility level of the RTV crystal after 24 h due to nucleus formation following its recrystallization. The crystallization of RTV was inhibited in the presence of both HPMC and chitosan, with induction times of 32 h and 24 h, respectively. This indicated that both chitosan and HPMC effectively inhibited the nucleation of RTV in the long term. It was also reported that the nucleation inhibition of drugs by polymers was attributed to the elimination and/or inhibition of the reorientation of the self-associated drug. Therefore, it can be assumed that the interaction between the carbonyl group of RTV and a proton of HPMC, as well as the amine group of RTV and a proton of chitosan, as observed in the NMR, FT-IR, and in silico study, can prevent nucleus formation in RTV by inhibiting the reorientation of RTV self-associations. The addition of the polymers was found to contribute to the molecular mobility suppression of the amorphous drug, leading to recrystallization inhibition. The results also show that the hydrogen bonds between RTV and the polymers (chitosan and HPMC) can suppress the molecular mobility of RTV, leading to the maintenance of the high supersaturation of RTV. The presence of chitosan not only contributed to recrystallization inhibition but also increased the amorphous solubility of RTV due to its solubilization. The presence of chitosan can maintain the molecular dispersion of RTV in a supersaturated solution. Despite the induction time of RTV in the chitosan solution being faster than in the HPMC solution, the RTV concentration in the chitosan solution was still greater after 62 h. This indicates that the inhibitory effect of chitosan on RTV crystal nucleation can be stronger than that of HPMC.

There is a need to discuss the difference in amorphous RTV solubility between the chitosan solution in water and the HPMC solution. A previous study reported that the formation of drug-rich droplets can affect the amorphous solubility of the drug, specifically when it is exceeded in an aqueous solution [6]. When RTV was dispersed in water, it rapidly formed drug-rich droplets, coarsened, and became micro-sized, leading to lower amorphous solubility, as observed in the HPMC solution. This indicated that HPMC did not significantly affect the molecular environment of molecularly dissolved RTV present in the aqueous solution or change amorphous RTV solubility. Moreover, a large amount of HPMC was distributed in the drug-rich droplets, leading to a reduction in amorphous drug solubility in HPMC solutions, and the crystal solubility of RTV was not achieved after 60 h. This is because the drug-rich droplets of RTV were probably still maintained, causing the crystallization inhibition of RTV. This result is potentially important because HPMC is commonly used as a polymer in amorphous solid dispersion formulations but diminishes the maximum achievable drug supersaturation of RTV. However, chitosan effectively suppressed the coarsening of the RTV-rich phase, leading to the maintenance of RTV molecular dispersion. Chitosan did not mix with the RTV-rich droplets but interacted with RTV in the aqueous solution through complex formation, which increased amorphous RTV solubility due to its solubilization effect. Previous studies reported that intermolecular interactions between the polymer and the drug in an aqueous medium through hydrogen bonding and hydrophobic interactions can contribute to the solubilization and crystallization inhibition of the drug [34,35]. These phenomena need to be confirmed by additional experiments in future studies.

## 5. Conclusions

The impact of chitosan on the supersaturation profiles of RTV and the mechanism for maintaining the supersaturation levels of a drug with a low recrystallization tendency in an aqueous solution was elucidated. The results showed that the solubility of amorphous RTV in the presence of chitosan was higher compared to its solubility with and without HPMC due to its solubilization effect. RTV did not rapidly crystallize at the beginning of the induction time measurement because it is a slow crystallizer. The induction time measurement also revealed that chitosan and HPMC suppressed RTV crystal nucleation by forming interactions with RTV in supersaturated solutions. This is because the interaction between the amine group of RTV and a proton of chitosan, as well as the carbonyl group of RTV and a proton of HPMC, leads to the long-term maintenance of RTV supersaturation. Based on the results, the inhibitory effect of chitosan on RTV crystal nucleation could be stronger than that of HPMC. This study also provides fundamental insight into pharmaceutical formulation development, where the addition of polymers is very important for the design of supersaturated formulations to optimize oral absorption, specifically for a drug with a molecular weight of over 500 g/mol.

## Figures and Tables

**Table 1:** Crystalline (S_c,RTV_) and amorphous (S_a,RTV_) solubility of RTV at 25 °C.

	Water	Chitosan Solution at 500 μg/mL	HPMC Solution at 500 μg/mL
S_c,RTV_	0.21 ± 0.04	0.23 ± 0.02	0.21 ± 0.03
S_a,RTV_	4.1 ± 0.95	7.47 ± 1.1	4.28 ± 1.06
S_a,RTV_/S_c,RTV_	19.52 ± 1.01	32.47 ± 1.1	20.38 ± 0.92

**Table 2:** Chemical shifts of protons from RTV, chitosan, and HPMC in the chitosan and HPMC solutions and the difference in the chemical shifts compared to those in the aqueous solution.

Sample	Chemical Shift (ppm)				Difference in the Chemical Shift (ppb)			
Peak	H8	H16	H21	H23	ΔH8	ΔH16	ΔH22	ΔH23
RTV	2.689	2.650	7.645	3.978				
RTV-HPMC	2.682	2.661	7.630	3.923	7	11	15	55
Peak	HIX		HXV		ΔHIX		ΔHXV	
HPMC	1.07		3.289					
RTV-HPMC	1.067		3.286		3		3	
Peak	H8	H16	H21	H29	ΔH8	ΔH16	ΔH21	ΔH29
RTV	2.631	2.65	7.645	2.865				
RTV-chitosan	2.619	2.656	7.644	2.854	12	6	1	11
Peak	Hb	He	Hf		ΔHb	ΔHe	ΔHf	
Chitosan	3.103	3.665	3.712					
RTV-chitosan	3.185	3.622	3.789		82	43	77	

**Table 3:** Result of viscosity measurement of each sample.

Sample	Viscosity (cps)
Water	4.22 ± 0.02
Chitosan solution	5.34 ± 0.04
HPMC solution	6.68 ± 0.04
RTV-chitosan solution	5.42 ± 0.06
RTV-HPMC solution	6.72 ± 0.03

## Data Availability

Not applicable.
